# The prototype description of a smartphone application ‘Maturo’: applying artificial intelligent technology to monitor the growth and maturation of youth

**DOI:** 10.3389/fpsyg.2026.1608796

**Published:** 2026-03-26

**Authors:** Ximing Shang, Jorge Arede, Pedro Couto, Rute Bastardo, Nuno Leite

**Affiliations:** 1 Beijing Normal-Hong Kong Baptist University, Zhuhai, China; 2 Department of Sports, Exercise and Health Sciences, University of Trás-os-Montes and Alto Douro, Vila Real, Portugal; 3 Research Center in Sport, Health, and Human Development (CIDESD), Vila Real, Portugal; 4 School of Education, Polytechnic Institute of Viseu, Viseu, Portugal; 5 Centre for Research and Technology of Agro-Environmental and Biological Sciences (CITAB), Inov4Agro, University of Trás-os-Montes and Alto Douro, Vila Real, Portugal; 6 School of Science and Technology, University of Trás-os-Montes and Alto Douro, Vila Real, Portugal

**Keywords:** growth and maturation, health application, monitoring, sport application, talent identification and development

## Abstract

**Introduction:**

Growth and maturation (GAM) monitoring is essential in youth sport and healthcare, particularly for talent identification, training prescription, and injury risk management. Recent advances in mobile technology have enabled the integration of artificial intelligence (AI), including computer vision (CV) and machine learning (ML), into widely accessible personal devices. However, practical and scalable tools for GAM monitoring remain limited.

**Methods:**

We developed a smartphone application, Maturo, using the Dart programming language. The app integrates automated data processing, AI-based analytical functions, and longitudinal growth tracking. The development process included user and expert advisory consultation, system architecture design, implementation of data security and privacy safeguards, interface prototyping, and translation of scientific models into a functional mobile platform.

**Results:**

Maturo enables users to monitor GAM through a smartphone, a device commonly available to athletes, parents, coaches, and healthcare professionals. The app supports automated maturation-related calculations, longitudinal data visualization, and structured reporting. The integration of AI-driven processing facilitates scalable and user-friendly monitoring without requiring advanced technical expertise.

**Discussion:**

This study represents the first phase of a broader project applying AI technologies to youth GAM monitoring. The development of Maturo demonstrates the feasibility of translating growth and maturation science into an accessible digital solution for sport and health settings. Future validation and real-world implementation studies are warranted to evaluate accuracy, usability, and impact.

## 1 Introduction

As the field of sports science continues to progress, the integration of cutting-edge technologies has become increasingly crucial to strengthen the long-term athlete development processes. One such transformative technology is AI, which has the potential to revolutionize the approach to talent (Faqihi and Miah, 2023). AI has already demonstrated its adaptability across various domains, including education, healthcare and business (Zhang et al., 2022). In the field of sports, AI-powered methods could be applied to analyze massive raw data, identify patterns, and make informed decisions that can significantly improve the accuracy and efficiency of Talent Identification and Development (TID) (Faqihi and Miah, 2023; Cossich et al., 2023). By using AI-driven algorithms, sports practitioners could now analyze a multitude of factors, such as an athlete’s physical attributes, technical, tactical and psychological to create comprehensive player profiles (Fu, 2020). This holistic approach to talent identification can help professionals uncover hidden gems, ensuring that the most promising athletes are identified and developed.

Adolescence represents a critical developmental window in youth sports, marked by substantial physiological and psychological changes (Güllich et al., 2022; Leite et al., 2021; Young et al., 2010). An accurate assessment of maturation processes is central to refining training routines and selection strategies, with the dual aim of enhancing performance (Cobley et al., 2009) and reducing health-related risks (Towlson et al., 2021). The recognition that variations in maturation timing exert significant influence on athletic performance, selection outcomes, training efficacy (Meylan et al., 2010) and injury proneness highlights the imperative for nuanced understanding of child athletes development (Arede, 2021). Despite this importance, the effectiveness of training and selection practices ultimately depends on the validity and reliability of the methods used to estimate GAM.

Conventional techniques for determining GAM status, such as radiographic methods to determine skeletal age (Chumela et al., 1989; Morris, 2003) and measuring sexual maturity based on Tanner stages (Malina et al., 2005; Rai et al., 2014), have limitations despite being useful. Radiography entails significant financial expenses, including the cost of X-ray imaging equipment, maintenance, and the need for trained radiologists to interpret the results. For instance, the average cost of a single X-ray scan ranges from $100 to $500 depending on the healthcare facility and region (Chumela et al., 1989; Morris, 2003). These costs can accumulate substantially in sports contexts, especially when repeated imaging is required for longitudinal monitoring.

Assessments of sexual maturity, in contrast, may be constrained by subjectivity, reliance on self-reported information, and privacy concerns when evaluating youth athletes (Fragoso et al., 2014; Matsudo and Matsudo, 1994). Although sonography provides a non-invasive alternative for estimating skeletal maturation (Guglielmi et al., 2009; Utczás et al., 2017), still it has limitations due to the array in the rate of bone development and occasional mistakes in identifying different phases of maturity.

While those traditional methods for assessing maturity have contributed substantially to GAM assessment, their limitations still necessitate to explore new approaches that are non-invasive, inventive, and cost-effective. The progress in statistical modeling, ML, and digital health technologies is revolutionizing the field of GAM research (Miotto et al., 2018). These advancements offer scientifically rigorous and practical methods that may be applied in several areas. As these technologies continue to evolve, they hold considerable potential for enhancing both the precision and scalability of GAM monitoring in young populations (Alber et al., 2019; Marsch, 2020).

The demand for non-intrusive and field-applicable methods of GAM monitoring has led to the development of several linear and nonlinear growth modeling techniques (Freeman et al., 1995; Grimm et al., 2011; Ostojic et al., 2014), allow for security data collection in real-world. Somatic methods derived from anthropometry have gained wide acceptance in soccer academies and other sports organizations for maturation indexing, determining the temporal distance from peak height velocity (PHV), and forecasting adult height, backed by standardized protocols from national regulatory bodies (Cumming et al., 2018; Fransen et al., 2021; Salter et al., 2022). Those methods still need the involvement of health experts or sports practitioners to control the estimation processing and explain the use of the GAM information to the players and guardians (coaches and parents).

Recent developments in mobile technologies have incorporated advanced AI methods such as CV, ML, and deep learning (DL) in personal devices. CV consists of a set of complex image recognition processes capable of detecting and classifying features from multimodal inputs. Combined with ML algorithms, CV can recognize human postures and joint points in images and subsequently process this information using DL algorithms to generate deeper insights (Khanal et al., 2022).

In parallel, several professional institutions have developed online tools for growth and maturity assessment, including the UK-WHO Growth Charts: 2–18 Years by the Royal College of Pediatrics and Child Health (RCPCH), the Maturity Status Calculator provided by the Office of Sport, NSW Government in Australia, and the Bioband Your Child tool from the University of Bath. These tools support the evaluation of growth patterns, biological maturity, and developmental stages in children and adolescents, offering valuable insights for healthcare professionals, educators, and re-searchers. But they are only with easy description of results (the biology age and growth status), without more deep info and guidance for players and coaches to use that. Also, the online calculator cannot automatically track the consistent GAM information and that is useful and good for longitude monitoring sport players. Other growth and maturity (GAM) monitoring tools like BoneXpert (which utilizes dorsopalmar radiographs) and BAUSport (which relies on ultrasound assessments) require professional health assistance, adding to their cost (ranging from $100 to $500 for radiographs/ultrasound assessments and software licenses) and complexity.

By addressing the limitations of traditional methods and offering scalable solutions for large-scale use (Baars et al., 2022; Bernaciková et al., 2022), AI technologies have the potential to transform growth monitoring (Guigas, 2017; Straun et al., 2024). Nonetheless, the transition to AI-based systems must proceed with caution, ensuring that their adoption is guided by rigorous validation to establish reliability and safety.

In response to these needs, our team developed an app with AI methods used in GAM of youth. This paper aims to describe a mobile app named Maturo that is designed to monitor the GAM of youth, that it could provide youth athletes and their guardians with awareness about the GAM information using an easy-to-use and low-cost tool that can be a good approach for helping to strength the long-term athlete development processes. Maturo integrates automated data capture, longitudinal growth tracking, advanced analytical models, and a user-friendly graphical interface, which could offer a practical and scalable solution for real-world youth sport settings.

## 2 Development description

The development of the Maturo app was the result of a collaborative initiative involving basketball and football coaches from Vila Real (Portugal) and researchers from the School of Science and Technology (ECT) and the Research Center in Sport, Health, and Human Development (CIDESD) at the Universidade de Trás-os-Montes e Alto Douro (UTAD). The current demo version of the app restricts access exclusively to healthcare professionals. Coaches and parents can only review data in collaboration with these professionals. Future iterations will implement a robust role-based access system, assigning tailored permissions to athletes, coaches, and healthcare providers.

### 2.1 Pre-development and design

User security and privacy are the primary considerations in our procedures of design and development of the mobile GAM monitor app, the login information screen of the login page is mandatory with the assigned unique username and password to access the app. Data from this app, used to manage personal information, data collection, usage and storage, are all stored locally on the smartphone without cloud storage, and we are keeping the use of Maturo only with limited group. This ensured compliance with privacy and security standards governing the collection, processing, and storage of personal data.

An expert advisory group comprising 2 parents, 6 coaches, 2 school educators, 3 sports science researchers, and 5 app developers worked as a team to make decisions regarding Maturo. The implications of the users’ needs analysis study findings and the effectiveness of identified GAM monitoring and visual data analytics were frequently discussed by the team. For instance, the inclusion of a graphical dashboard to visually track GAM over time was proposed by the coaches and supported by parents, highlighting the importance of user-friendly interfaces for non-technical users. Similarly, the educators emphasized the integration of age-appropriate language and educational resources within the app, ensuring that young athletes and their families could easily interpret the data.

**Figure 1:**
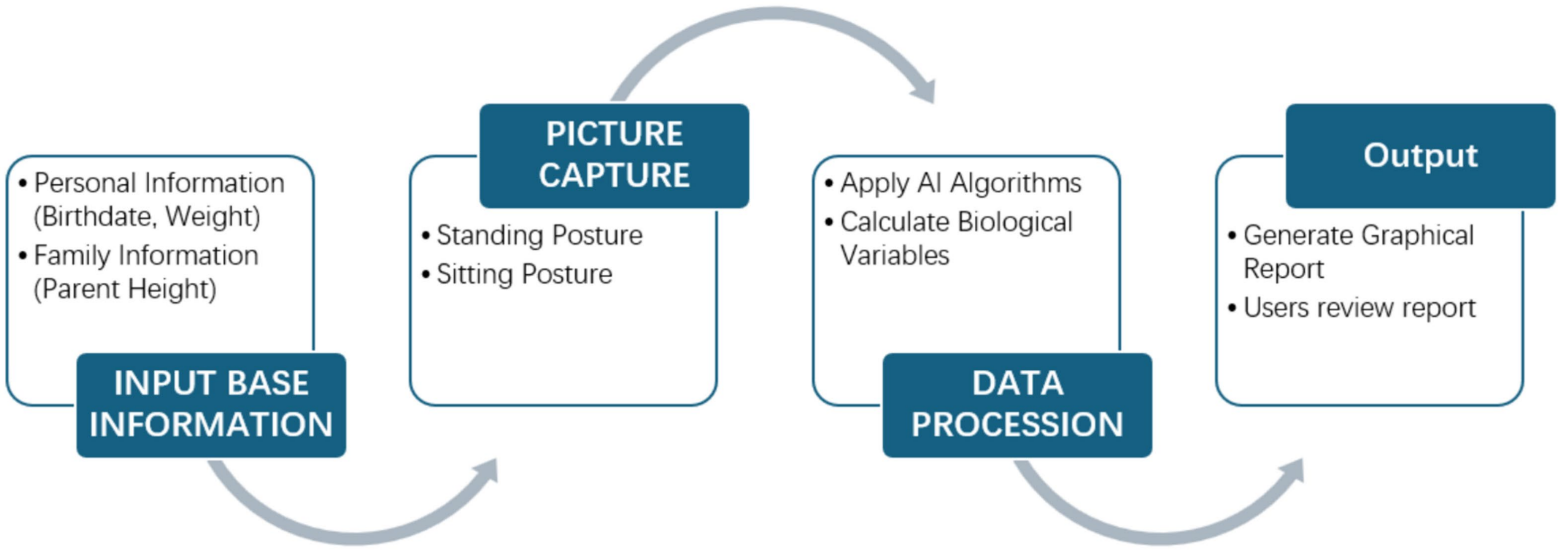
Flowchart of the Maturo software process.

Although the advisory group represented key stakeholders in youth sport and education, we acknowledge that clinical stakeholders were not included in the initial prototype phase. As Maturo is currently an exploratory proof-of-concept application, early development focused on sport-science and technology perspectives. Nevertheless, we recognize the critical importance of pediatric and clinical expertise for ensuring safe interpretation of maturation data and alignment with health-monitoring best practices. Therefore, the future development phases will formally incorporate pediatricians, sports-medicine specialists, and data-protection professionals to guide medical validation, refine the interpretation of maturation stages, and strengthen ethical and clinical governance (Figure 1). The application follows GDPR principles, ensuring data minimization, encryption, and role-based access control. The current version restricts access to healthcare professionals, and future updates will include a robust permission system differentiating access for athletes, coaches, and healthcare professionals.

### 2.2 Development of Maturo

Maturo is a mobile app created with the Dart programming language (Dart Team at Google. (2024). Dart Programming Language). The program leverages widely available technology by operating a smartphone with a digital camera and a brief questionnaire and two images of the user. Maturo’s core principle is to expand the availability of sophisticated growth monitoring tools by using AI models.

### 2.3 Functionalities and features

#### 2.3.1 User interface and onboarding

**Figure 2:**
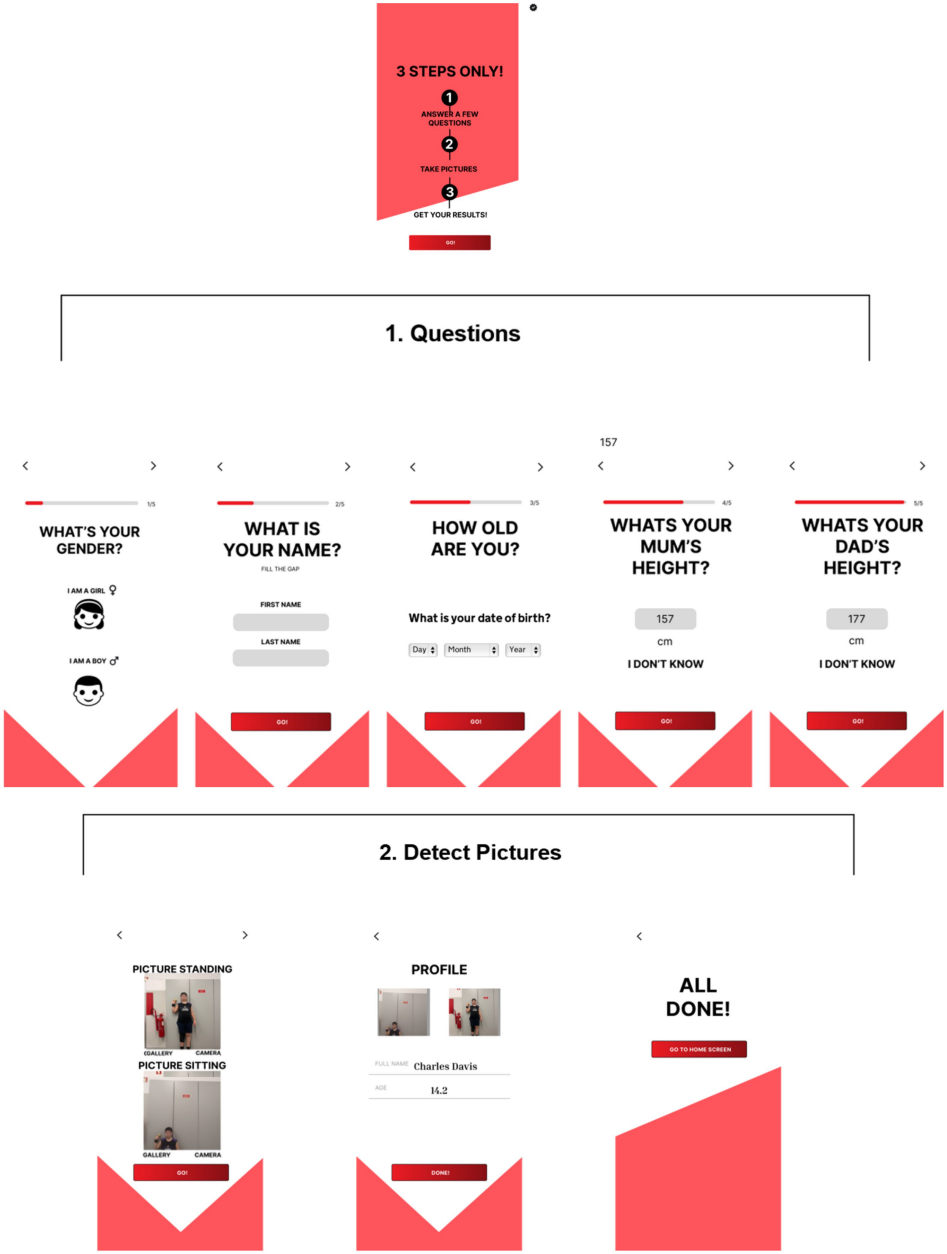
The workflow of GAM monitoring: (1) Question input; (2) pictures capture; and the profile creation.

The app interface is designed with the intent to provide easy and intuitive interaction for all users. Initiation encompasses the following (Figure 2):

Question input: A concise yet comprehensive survey is conducted to begin the process, gathering information on sports institutions, name, birth date, gender and parents’ height to create an initial profile of the athlete. Parents are explicitly informed and must give consent before providing their height data. Without this consent, the information will not be stored or used by the application, ensuring GDPR compliance.
Pictures capture: The Maturo app uses the smartphone’s camera to capture images of the athlete in both standing and sitting postures (Figure 1). While most smartphone cameras are compatible with the app, there are certain minimum quality requirements to ensure accurate analysis. The camera must support at least 8 megapixels and a frame rate of 30 fps (frames per second) to provide clear, high-resolution images suitable for the app’s analytical algorithms. These specifications help ensure consistency and precision in measurements derived from the captured images. The demo version of Maturo restricts access exclusively to healthcare professionals. Parents and coaches can only review data alongside these professionals. Future versions will implement a robust permission system, ensuring different access levels for athletes, coaches, and healthcare professionals.

#### 2.3.2 Maturation assessment and monitoring

**Figure 3:**
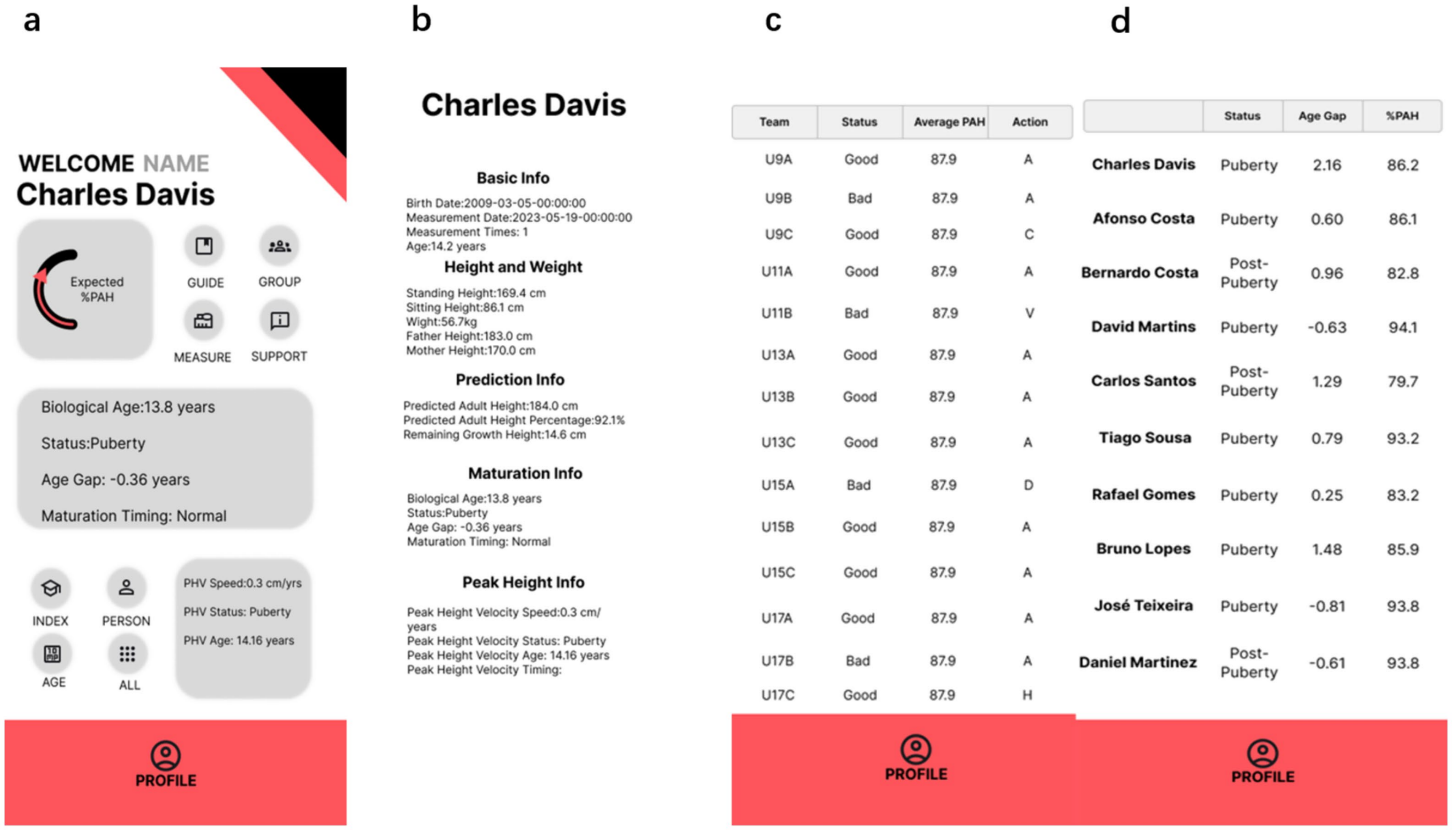
Visual report of individual and team example from Maturo. **(a)** Person general page; **(b)** person detail page; **(c)** team general page 1; **(d)** team general page 2.

Maturo’s primary functionality lies in its advanced capabilities for maturation assessment and ongoing monitoring. The diagnostic system integrates responses to a validated questionnaire and biometric data, such as height, weight, and pubertal characteristics, to categorize athletes into maturity groups (e.g., early or late-maturing). This approach is grounded in established methodologies, such as the Khamis-Roche method and its derivatives, which have been widely used for non-invasive assessment of biological age and growth trajectories. By combining these validated methods with real-time data processing, Maturo enables more precise grouping of athletes, aligning with evidence-based practices in sports science. As shown in Figure 3, which illustrates athletes’ growth trajectories, maturation indicators, and other key metrics. These graphical outputs enable sports professionals to easily identify developmental patterns and detect irregularities in progress.

#### 2.3.3 GAM and training recommendations

**Figure 4:**
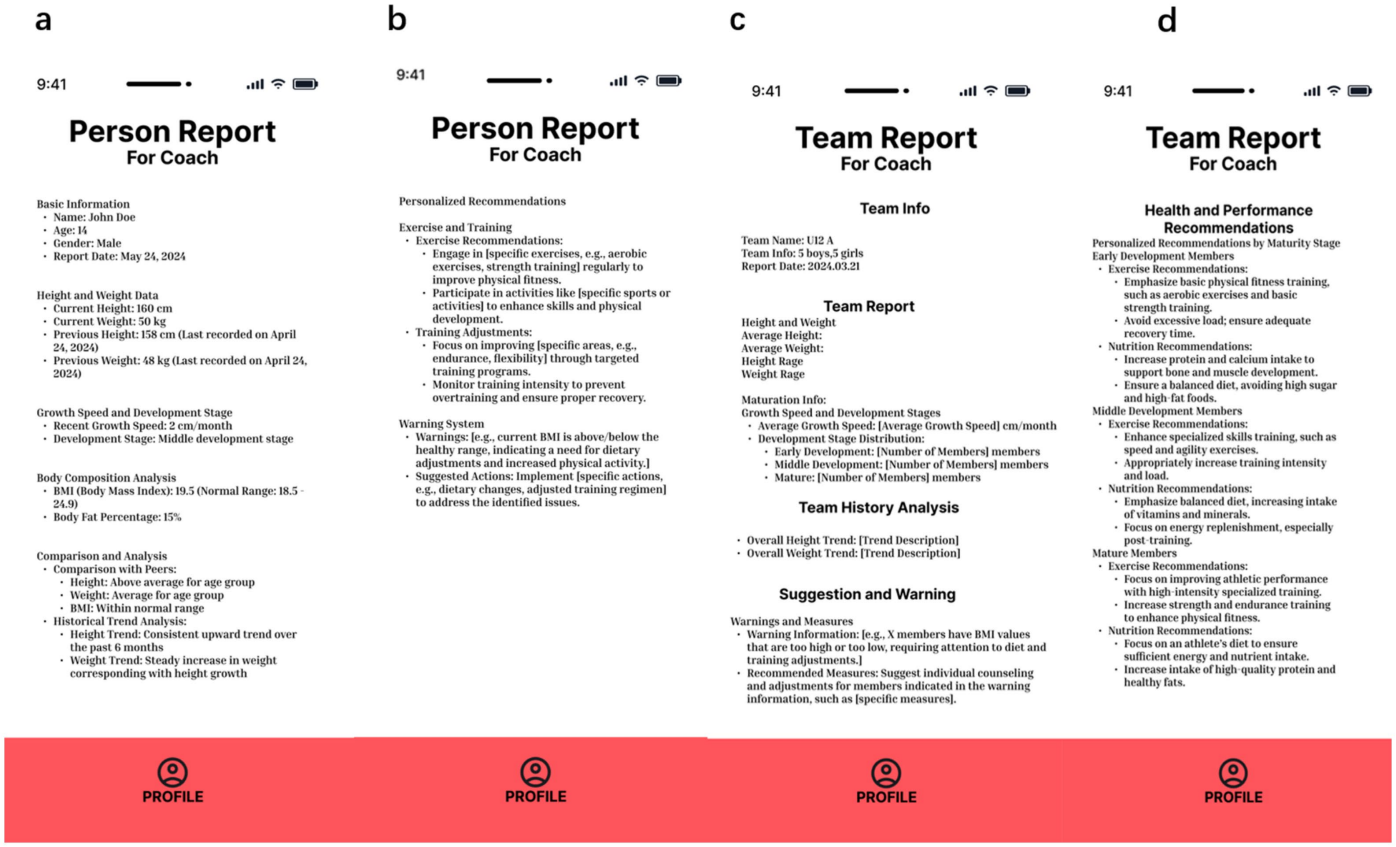
Detailed information and suggestion of individual and team examples from Maturo. **(a)** Person general information; **(b)** person recommendation; **(c)** team general information; **(d)** team recommendation.

Maturo’s adaptive features extend beyond basic monitoring by providing actionable GAM insights and training recommendations (Figure 4): The system classifies athletes based on their maturation phase (timing and status), enabling the creation of tailored training plans that align with specific developmental milestones and the unique needs of each group. Additionally, Maturo is designed to support advisory coaches in providing personalized guidance by leveraging its data-driven features. The system integrates individual athlete profiles, comprising maturity status, growth trajectory, and biometric data—to generate tailored notifications that align with evidence-based guidelines for youth athletic development (Towlson et al., 2021; Arede, 2021). For instance, based on indicators of growth spurts or delayed development, the app offers recommendations to adjust training intensity, duration, and recovery protocols. These features aim to minimize the risk of overtraining and injuries while fostering a safer and more efficient progression in athletic performance. By integrating validated prediction models for biological age and peak height velocity (PHV) (Meylan et al., 2010), the app enhances the advisory coach’s ability to individualize training programs grounded in scientific principles.

### 2.4 The applying of Maturo

#### 2.4.1 Comparison procedure of expert method and Maturo

During the period from October 2023 to December 2024, we assessed the accuracy and consistency of Maturo in serval time. Those evaluations were conducted on total of 144 adolescent athletes (76 males and 68 females) aged 10 to 17 years, comparing Maturo’s estimates to traditional expert evaluations.

The 144 adolescent athletes were recruited from local sports clubs and school-based sports programmes using predefined inclusion and exclusion criteria. The inclusion criteria required participants to be healthy adolescents aged 11–17 years, actively participating in organized sports for at least one year, and free from any conditions affecting growth assessment. Exclusion criteria included recent musculoskeletal injuries, chronic medical conditions, and incomplete anthropometric records.

To ensure compliance with ethical standards, written informed consent was obtained from all participants and their legal guardians. The evaluation involved both expert assessments and Maturo’s automated analysis at the same time.

Initially, athletes underwent a conventional evaluation where 3 operators were involved. Two operators measured weight (with shoes removed) to the nearest 0.1 kg using a SECA scale (model Clara 803, Hamburg, Germany). They also measured standing height and sitting height (with shoes removed) to the nearest 0.1 cm using a stadiometer (SECA model 213, Hamburg, Germany). The third operator recorded the data and measurements manually. This method involved human measurement and analysis. Operators measured participants’ anthropometric data (weight, standing height, and sitting height) and manually analyzed the data to forecast MO and PAH. The experts analyzed the data by calculating an objective maturity offset using standardized predictive equations based on the athletes’ height, weight, and sitting height.

Following the expert evaluation, participants underwent an initial assessment using the Maturo app. The setup involved ensuring the unit was horizontal and level using the spirit level feature on an iPhone. The phone was positioned 2 meters from the background wall and 1 meter from the ground. One operator assisted athletes in completing a questionnaire and manually recorded their weight after measuring each participant using a standard scale (Seca, model Clara 803®, Hamburg, Germany). The operator then took photos to measure the athletes’ height, as illustrated in Figure 2. This method employed the Maturo app to record anthropometric data.

There is no missing data occurred during the anthropometric and maturity assessments. All height, sitting height, and age records were fully collected during each evaluation session.

#### 2.4.2 Comparison procedure of expert method and Maturo

By comparison, Maturo utilized the same anthropometric data but automated the analysis, significantly reducing evaluation time (Table 1). The time required for data collection and result presentation differed notably between the two methods: Maturo significantly reduces evaluation time, both in data entry (3–7 s vs. 5–10 min) and result presentation (real-time vs. one week delay).

**Table 1:** Data collection, result presentation and labor requied.

Measurement method	Measurement time	Result processing time	Required personnel	Required equipment	Efficiency
Expert method	5–10 min	1 week	3	Weight scale, stadiometer, notebook	Manual processing
Maturo software	3–7 s	Real-time	1	Weight scale, smartphone	Instant results

**Figure 5:**
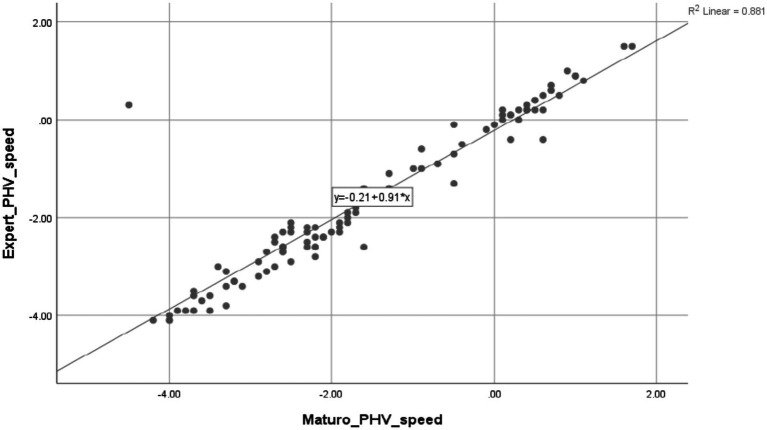
Intraclass correlations and scatterplots for estimates of PHV speed derived from the Maturo software and expert protocol.

**Figure 6:**
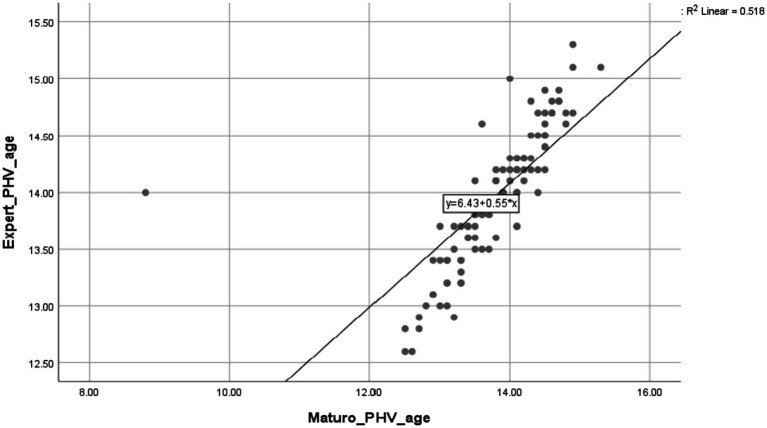
Intraclass correlations and scatterplots for estimates of PHV age derived from the Maturo software and expert protocol.

In order to thoroughly assess the level of agreement between Maturo’s automated predictions and the results obtained from expert methods, intraclass correlation coefficients (ICCs) were computed. The ICCs were used to evaluate the strength and direction of the relationships between the automated estimations of maturation and the reference method, using a combination of mixed effects and absolute agreement. The results may be seen in Table 2. The Maturo software exhibited high reliability, with ICC values exceeding 0.9 for PHV speed, indicating excellent agreement. The smallest mean differences and Technical Error of Measurement (TEM) values were observed that suggesting minimal discrepancies between the methods. However, the ICC for PHV age was 0.673 (95% CI: 0.556–0.777), indicating moderate reliability. This suggests that further refinement may be needed for this variable when using the Maturo. Scatter plots illustrating the correlations between the automated method (Maturo software) and the expert method are depicted in Figures 5, 6. These plots visually reinforce the high degree of agreement between the methods.

**Table 2:** Comparison of methods for estimating biological age against the expert method for the youth.

Measurement comparison	Mean (SD)	ICC (95% CI)	A. TEM	R. TEM
PHV SPEED _Expert vs. Maturo	−0.09 (0.47)	0.946 (0.910–0.962)	0.09	0.80
PHV AGE _Expert vs. Maturo	0.17 (0.54)	0.673 (0.556–0.777)	0.17	0.76

ICC = Intraclass correlation, A. TEM = Absolute Technical Error of Measurement, R. TEM = Relative Technical Error of Measurement.

We have cross-tabulation tables of maturity classifications between the Maturo software and expert assessments (Table 3). The table categorizes athletes into three maturation stages: Pre-Pubertal, Pubertal, and Post-Pubertal, based on both assessment methods. Among the 52 athletes classified as Pre-Pubertal by experts, and Experts identified 39 athletes as Post-Pubertal, and the Maturo software classified the same 39 athletes correctly, showing full agreement in this category. For the 50 athletes classified as Pubertal by experts, Maturo software correctly classified 47, with only three misclassifications into the Pre-Pubertal category. Out of 144 athletes, 138 were classified identically by both methods, suggesting strong alignment between the expert method and Maturo software.

**Table 3:** The crosstabulation of maturation status between expert method and Maturo software.

	Maturo software				
	Count	Post-pubertal	Pre-pubertal	Pubertal	Total
Expert method	Post-pubertal	39	0	0	39
	Pre-pubertal	0	52	0	52
	Pubertal	0	3	47	50
	Total	39	55	47	144

## 3 Technological overview

Maturo has been designed to accommodate a broad range of users, from young athletes to adults, and includes an educational portal that supports coaches, guardians, and teenagers in understanding GAM information. The application is engineered as a mobile application, utilizing the flexibility of Dart to provide a distinct experience on Android platforms, enabling smooth integration of CV and ML model components. This strategic choice not only improves user accessibility but also could have a fast development cycle and maintenance operations.

The user interface follows contemporary principles from Material Design (Version 3, Google, 2024), to provide responsive layouts, straightforward navigation, and captivating animations. The app employs a simplified navigation system with clearly labeled buttons and intuitive workflows, reducing cognitive load for users with limited technical expertise (e.g., younger athletes, parents unfamiliar with technology). And Material Design’s framework emphasizes contrast, legible typography, and accessible color schemes, which are particularly beneficial for users with visual impairments or color blindness. These features aim to enhance usability and encourage active user participation (Ahmadi and Kong, 2012).

Maturo applies YOLO (You Only Look Once) v7 algorithms to enhance the precision and efficiency of its maturation assessments, and all algorithm and the following information processing are run locally on the device. YOLO v7 is notable for its architecture based on F-CNN (Fully Connected Neural Network), which enables faster and more accurate deductions (Wang et al., 2023). The image processing pipeline begins with preprocessing steps such as noise reduction, image normalization, and segmentation to extract critical features from anthropometric images. This ensures that data input quality is consistent and optimized for subsequent analysis. The DL component, built on convolutional neural networks (CNN), processes these extracted features to identify patterns indicative of biological maturation (based on the Khamis-Roche method). All application data is stored locally on the user’s device, without reliance on external servers. This approach eliminates concerns related to server-side data processing and ensures that all personal information remains under the user’s control.

When an athlete uploads an image, the YOLO model identifies key growth markers, such as standing height, sitting height, and limb ratios, through bounding box detection. These detected features are then processed to calculate critical metrics, which feed into the app’s biological age estimation model. The pre-trained YOLO model used in this app was fine-tuned with a custom dataset of 1775 manually tagged images of youth athletes, ensuring its relevance to GAM assessment. The app processes images locally on the user’s device, ensuring both real-time feedback and data security. By using multiple layers of feature extraction and classification, the system can estimate biological age and predict growth trajectories with high accuracy.

To ensure adaptability across diverse populations, the algorithms incorporate transfer learning, enabling the model to fine-tune its predictions based on new data while leveraging knowledge from the original training set. This combination of advanced image processing and DL enables Maturo to automate complex assessments, minimizing the need for manual intervention while maintaining consistency and accuracy.

## 4 Discussion

This study addresses the development and implementation of the Maturo method, highlighting its comparative advantages in real-time GAM tracking. Maturo is an innovative program that uses AI techniques (CV and ML) to automate the assessment and training design in youth sports. By including several data and recognizing individual differences, these models provide a strong basis for assessing maturation using non-invasive methods. In addition, the rise of ML has brought up new techniques for predicting growth and categorizing maturity (Dallora et al., 2019).

Monitoring GAM has become an essential component of contemporary youth sport systems, which helps the coaches to offer tailored programs that align with players’ physical conditions, tactical requirements, and stages of development. This approach maximizes player development while minimizing the risk of injuries. This is particularly relevant for youth players, when rapid growth spurts during adolescence are believed to significantly increase the risk of injuries among young athletes (Johnson et al., 2022). Therefore, implementing scientifically monitoring during this critical period becomes essential. A key takeaway from Salter’s research is the emphasis on the need for accurate biological maturity estimation in the context of soccer academies, particularly for improving injury prevention strategies and tailoring developmental programs for young athletes. However, the consistent application of such practices is often constrained by limited resources, time, and staff expertise (Salter et al., 2022).

Several professional institutions have introduced online calculators for estimating growth and maturity, yet many of these tools require institutional licenses or specialized access, making them inaccessible to parents, coaches, or grassroots sport programs. This barrier reduces their utility in community sport settings where affordability, simplicity, and frequent monitoring are essential.

Maturo differs fundamentally from these systems by functioning solely through a smartphone application, eliminating the need for costly external devices. This significant reduction in equipment costs makes Maturo a far more cost-effective solution. Furthermore, the ease of use associated with an APP means that regular, even weekly, assessments are feasible without requiring extensive setup or specialized training. This affordability and simplicity are crucial for enabling consistent monitoring and timely interventions, ensuring athletes’ developmental needs are met promptly and efficiently.

Beyond sports context, Maturo holds considerable potential for pediatric growth monitoring, offering critical insights for medical professionals and parents alike. Maturo may enable early interventions by properly identifying children who are developing either earlier or later than expected standards, based on precise predictions of development speed. Early treatments are essential for addressing any health or developmental problems, enabling customized assistance and maximizing growth trajectories.

The development of Maturo is grounded in established theoretical perspectives on youth growth, biological maturation, and technology-supported assessment. First, the estimation of biological maturation draws on widely adopted growth and maturation models, particularly the maturity offset and predicted adult height frameworks proposed by Mirwald et al. (2002) and later refined by Malina et al. (2005). These models highlight the nonlinear and individually variable nature of adolescent growth, emphasizing the importance of accessible and repeatable assessment tools for youth sport and health settings.

From a developmental perspective, monitoring biological maturation is central to long-term athlete development pathways. The Long-Term Athlete Development (LTAD) framework (Balyi, 2004) and the Developmental Model of Sport Participation (Côté et al., 2003) both underline the need to align training with biological, rather than chronological, maturation. These models provide a conceptual rationale for tools that can inform coaches, parents, and young athletes about maturation timing in a non-invasive and user-friendly manner.

Given that digital tools increasingly support youth health management, the adoption and use of Maturo can also be situated within technology acceptance and human–computer interaction theories. Constructs from the Technology Acceptance Model (Davis, 1989) and related digital health engagement literature emphasize perceived usefulness, ease of use, and trust as key determinants for the uptake of health monitoring applications by adolescents and their caregivers. These frameworks explain why intuitive design, device accessibility, and transparent data presentation are necessary components of a maturation assessment tool.

This study was conducted in accordance with established ethical standards for research involving minors and was approved by the institutional ethics committee. All participants and their legal guardians provided informed consent prior to data collection. To ensure participant autonomy and comprehension, procedures were explained in age-appropriate language, and assent was obtained from adolescents in addition to parental consent.

Given that the present work represents an initial exploratory validation of the automated maturation assessment approach, several precautions were taken to safeguard the participants’ rights and privacy. All data collected through the study were anonymized prior to analysis, and no personally identifiable information was stored within the system or used for any stage of model development. Data collection and interpretation were supervised by a multidisciplinary team including coaches, sport science specialists, and computer science researchers to minimize risks associated with measurement, interpretation, and data handling.

Recognizing the importance of broader ethical governance in digital health tools, especially those involving minors, we acknowledge that additional expertise is essential for future development. To address the concerns surrounding autonomy, informed consent, data protection, and responsible interpretation, future versions of the application will be guided by an interdisciplinary advisory board consisting of pediatricians, data protection specialists, and ethics scholars. This advisory structure will support the refinement of data governance practices, ensure compliance with evolving international regulations, and strengthen user protection as the system transitions from research prototype to real-world implementation.

The present manuscript represents the first stage of a broader project on GAM monitoring and has already been piloted with more than 200 youth athletes to evaluate the usability of the app in sport environments. Given that PHV age is a second-order temporal feature derived from individual growth-velocity curves, its estimation from cross-sectional anthropometric data inherently results in moderate reliability. This structural limitation has been consistently documented in previous maturation research, where accurate identification of the maximum height-velocity inflection point typically requires longitudinal measurements.

In addition to the maturation prediction approaches most commonly cited in the international literature, it is important to acknowledge that alternative models and population-specific investigations have been developed in different research contexts. Notably, studies conducted in Brazilian youth populations have contributed valuable insights into biological maturation assessment and pubertal prediction, emphasizing the influence of demographic, socioeconomic, and developmental variability on growth trajectories (Mm et al., 2015; de Almeida-Neto et al., 2025; Cabral et al., 2013). These investigations highlight that maturation-related indicators may exhibit population-specific characteristics and that predictive accuracy can vary across cohorts with distinct biological and environmental profiles.

Recognizing this body of work reinforces the need for caution when generalizing maturation estimates across diverse youth populations. In this context, the Maturo application is not intended to replace established methods or to assume universal predictive validity, but rather to provide a practical and scalable tool that integrates established growth frameworks while remaining adaptable to future population-specific calibration. Future validation studies will explicitly explore cross-cultural performance and model generalizability by incorporating datasets from different regions and developmental contexts.

In this exploratory prototype study, correlation analyses were used deliberately to quantify measurement association rather than causal dependency. Because Maturo is still in an early development phase and the sample size is limited, dependency modeling or regression analysis was not conducted to avoid over-interpretation of preliminary results. Future studies with larger multi-timepoint datasets and longitudinal follow-up will incorporate predictive modeling, advanced calibration approaches, and more robust curve-reconstruction techniques. A separate validation study comparing Maturo estimations with expert assessments has been published elsewhere and provides a more comprehensive analysis of measurement accuracy.

The present manuscript is explicitly positioned as a developmental description of the design rationale, functional architecture, and applied feasibility of the Maturo smartphone application for growth and maturation (GAM) monitoring in youth populations. Although selected quantitative indicators, such as intraclass correlation coefficients, technical error of measurement, and cross-tabulation of maturity classifications, are reported, these analyses are included solely to demonstrate system operability and practical agreement, rather than to provide comprehensive algorithmic validation. A separate, previously published study has provided a detailed outcome-level validation of Maturo-derived maturation estimates against expert-based assessments, focusing on measurement agreement and applied reliability in youth sport settings (Shang et al., 2025).

A further limitation relates to the current prototype’s reliance on a small development team, which constrains the speed and scope of feature expansion and technical refinement. As a low-cost application, Maturo does not yet provide all functionalities seen in larger, resource-intensive commercial systems, and continued development will require sustained collaboration and external funding. These limitations highlight the necessity of future studies assessing long-term usability, ecological validity, and effectiveness across diverse real-world training and health-monitoring settings.

## 5 Conclusion

The Maturo app could provide automated monitoring of key physical characteristics, including biological age, growth timing, and peak height velocity. As an early-stage prototype, the current version demonstrates the feasibility of integrating computer-vision techniques and anthropometric prediction models into a practical tool for youth sport and health contexts. While preliminary findings suggest that Maturo may offer a low-cost and user-friendly alternative to traditional assessment methods, its potential applicability in underserved or resource-limited regions remains hypothetical, as this study did not directly evaluate such settings. Therefore, further research, including multi-site testing, cross-cultural validation, and long-term real-world deployment studies, is required to determine the app’s effectiveness, usability, and broader accessibility.

## Data Availability

The raw data supporting the conclusions of this article will be made available by the authors, without undue reservation.
