# Correction: The prototype description of a smartphone application ‘Maturo': applying artificial intelligent technology to monitor the growth and maturation of youth

**DOI:** 10.3389/fpsyg.2026.1856846

**Published:** 2026-05-07

**Authors:** Ximing Shang, Jorge Arede, Pedro Couto, Rute Bastardo, Nuno Leite

**Affiliations:** 1 Beijing Normal-Hong Kong Baptist University, Zhuhai, China; 2 Department of Sports, Exercise and Health Sciences, University of Trás-os-Montes and Alto Douro, Vila Real, Portugal; 3 Research Center in Sport, Health, and Human Development (CIDESD), Vila Real, Portugal; 4 School of Education, Polytechnic Institute of Viseu, Viseu, Portugal; 5 Centre for Research and Technology of Agro-Environmental and Biological Sciences (CITAB), Inov^4^Agro, University of Trás-os-Montes and Alto Douro, Vila Real, Portugal; 6 School of Science and Technology, University of Trás-os-Montes and Alto Douro, Vila Real, Portugal

**Keywords:** growth and maturation, health application, monitoring, sport application, talent identification and development

In the published article, an incorrect grant number was provided UI/04045 in the funding statement. The correct number is UID/04045/2025 (https://doi.org/10.54499/UID/04045/2025), UID/PRR/04045/2025 (https://doi.org/10.54499/UID/PRR/04045/2025), and UID/PRR2/04045/2025 (https://doi.org/10.54499/UID/PRR2/04045/2025).

The funding statement was incorrectly stated as: The author(s) declared that financial support was received for this work and/or its publication. This work was funded by National Funds by FCT - Foundation for Science and Technology under the following project UI/04045. Ximing Shang was funded by China Scholarship Council, grant number 202207920001.

The correct statement is appears below:

The author(s) declared that financial support was received for this work and/or its publication. This work was funded by National Funds by FCT - Foundation for Science and Technology, under the projects UID/04045/2025 (https://doi.org/10.54499/UID/04045/2025), UID/PRR/04045/2025 (https://doi.org/10.54499/UID/PRR/04045/2025), and UID/PRR2/04045/2025 (https://doi.org/10.54499/UID/PRR2/04045/2025). Ximing Shang was funded by China Scholarship Council, grant number 202207920001.

The original version of this article has been updated.

